# Management of neonatal pulmonary hypertension-a survey of neonatal intensive care units in India

**DOI:** 10.1186/s12887-023-03964-9

**Published:** 2023-03-31

**Authors:** Pari Singh, Sujata Deshpande, Rema Nagpal, Reema Garegrat, Samir Gupta, Pradeep Suryawanshi

**Affiliations:** 1 Bharati Vidyapeeth Deemed University, Pune, India; 2 B. J. Medical College & Sassoon Hospital, Pune, India; 3 Durham University, Durham, UK

**Keywords:** Persistent pulmonary hypertension of newborn, Indian survey, Targeted neonatal echocardiography, Pulmonary vasodilators, Inhaled nitric oxide, Inotropes

## Abstract

**Background:**

Persistent pulmonary hypertension of the newborn (PPHN) is a common neonatal condition associated with significant morbidity and mortality. First-line diagnostic and treatment options such as echocardiography and inhaled nitric oxide (iNO) are not routinely available in resource limited settings and alternative treatment modalities need to be utilized. This study was conducted to assess current diagnostic and management strategies used for PPHN in Indian neonatal intensive care units (NICUs).

**Methods:**

A questionnaire in multiple choice question format was sent to practising neonatologists in India via an online survey tool between July to August 2021. Information pertaining to demographic data, diagnostic criteria and management strategies of PPHN was requested. The responses were collated and information processed.

**Results:**

There were 118 respondent NICUs (response rate 74%). The majority of neonatal units (65%) admitted an average of 1–3 patients of PPHN per month. Targeted neonatal echocardiography (TnECHO) was practised in 80% of the units. Most common management strategies being followed were pulmonary vasodilators (88.1%), inotropes (85.6%), conventional ventilation (68.6%) and high frequency ventilation (59.3%). The most preferred pulmonary vasodilator was sildenafil (79%) and inotropic agent was milrinone (32%). Only 25% of respondents reported use of iNO. None of the participating units used extracorporeal membrane oxygenation.

**Conclusion:**

We found wide variability in management practices of PPHN across Indian NICUs. Non-selective pulmonary vasodilators are more widely used than iNO. There is an urgent need for structured TnECHO training programs and evidence based national guidelines for standardized management of PPHN as per availability of resources in India. Additional research on low cost alternative therapies to iNO in Indian settings might be helpful.

## Background

Persistent pulmonary hypertension of the newborn (PPHN) is a disorder of failure of circulatory transition of the newborn, where the high fetal pulmonary pressures do not reduce to normal levels after birth. Decreased pulmonary blood flow and increased right to left shunt across patent foramen ovale or ductus arteriosus leads to hypoxia, decreased cardiac output and complications due to poor tissue oxygenation [1, 2]. Despite advances in treatment modalities, the morbidity and mortality of this condition has remained high, especially in developing countries [3–5].

Pathophysiology of this disease is complex, involving various pathways such as cyclic AMP, cyclic GMP and endothelin, which in turn affect pulmonary vascular tone. The interventions such as nitric oxide, sildenafil, milrinone, prostacyclin and bosentan utilize these pathways to manage PPHN [6]. Multiple treatment modalities have been studied and are available for the management of this condition. Over the years, there has been advent of new treatment options and there is wide variation in management strategies used in neonatal intensive care units (NICUs) across the world [7].

In a developing country like India, diagnostic and treatment modalities of choice for PPHN, such as echocardiography and inhaled nitric oxide (iNO) may not be easily available and the treating neonatal physician has to resort to low cost alternatives [8]. Furthermore, availability of resources is not uniform across different types of health care facilities in India. Management practices are therefore likely to vary across different NICU settings. This cross-sectional observational study was designed to understand the current preferences and available options for management of PPHN in Indian NICUs, and whether any barriers exist, for optimum management of this condition. The current Indian data for this is scarce. We are hopeful that the observations from this study may help to establish clinical practice guidelines for Indian NICUs tailored as per available resources, as well as highlight areas for future research and innovations for low cost alternative treatment modalities.

## Methods

This cross-sectional study was conducted at a tertiary care neonatal center of a teaching hospital of India, using a survey questionnaire comprising of 38 items, between July to August 2021. The questionnaire requested information pertaining to demographic data, diagnostic criteria and management strategies of PPHN in Indian NICUs, in a multiple choice question format. Approval was obtained from the institutional ethics committee. Before circulation, the questionnaire was emailed for review to five practising neonatal physicians in different parts of the country and the content was finalized after incorporating their inputs.

An online survey tool (‘Google form’—https://www.google.com/forms/about/) was drafted from the questionnaire content. The survey link was tested for feasibility by two neonatologists and neonatal fellows. After further modification, the final version of the questionnaire was disseminated to practising neonatologists in the country via email or text message with a link to the questionnaire. Weekly reminders were sent and the survey was closed after a period of one month. Contact details of neonatologists were obtained from directories of Neonatology chapter of Indian Academy of Paediatrics and National Neonatology Forum of India. A brief background of the purpose of the survey was provided with the link. Consent was implied by participation in the survey and no incentives were offered for taking part in the survey. The data received was kept confidential. In case of multiple entries from the same unit, data provided by the senior neonatologist was considered final for that unit.

## Statistical analysis

Results obtained were formatted into an excel sheet and descriptive analysis was done using the SPSS V. 25 software for statistical analysis. Qualitative data was analysed as frequencies and percentages, and quantitative data was presented as mean, standard deviation, or median and range.

## Results

### Characteristics of survey participants

**Fig. 1:**
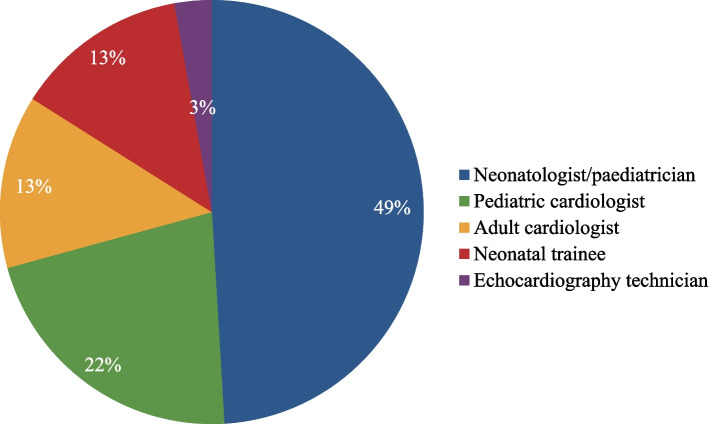
Category of personnel performing echocardiography for PPHN

A total of 118 neonatal units across the country responded to the survey questionnaire (response rate 74%). Of these, 92% were formally trained in neonatology and 97% were practicing in-patient newborn care. The experience of the respondents in the field of neonatology varied and is outlined in Table 1. Approximately 38% responses were from neonatal units attached to medical colleges, and 62% were from non- teaching units. Around 22% were government institutions while 78% were privately run hospitals. Other characteristics of the participating units are depicted in Table 1. Majority of the units (64%) admitted an average of 1–3 patients of PPHN per month. Echocardiography facility was available in 90% of the units and was performed by neonatologists, pediatricians or cardiologists (see Fig. 1). Targeted neonatal echocardiography (TnECHO) for PPHN was being practised in 80% of the units. Experience and training of physicians practising TnECHO is depicted in Table 1.

**Table 1:** Characteristics of survey participants

Characteristic	Responses N	Percentage %
**Formal training in Neonatology (*n* = 118)**	108	91.5
**Neonatal in-patient practice (*n* = 118)**	115	97.4
**Duration of experience in neonatology (*n* = 118)**		
< 3 years	16	13.6
3–5 years	25	21.1
5–10 years	31	26.3
> 10 years	46	39
**Type of admissions (*n* = 118)**		
Predominantly inborn	16	13.6
Predominantly outborn	26	22
Both	76	64.4
**NICU bed capacity (*n* = 118)**		
< 10 beds	23	19.5
10–30 beds	75	63.6
30–50 beds	15	12.7
> 50 beds	05	4.2
**Total number of NICU admissions (*n* = 118)**		
< 50/month	61	51.7
50–100/month	39	33
100–200/month	14	11.9
> 200/month	04	3.4
**Number of patients with PPHN per month (*n* = 118)**		
1–3	76	64.4
4–6	30	25.4
> 6	12	10.2
**Practice of routine TnECHO (*n* = 118)**	94	79.7
**Echocardiography performed by (*n* = 106)**^a^		
Neonatologist/ paediatrician	52	49.1
Pediatric cardiologist	23	21.7
Adult cardiologist	14	13.2
Neonatal trainee	14	13.2
Echocardiography technician	03	2.8
**Individual experience of people performing TnECHO (*n* = 94)**^a^		
< 1 year	36	38.3
1–3 years	33	35.1
4–5 years	12	12.8
> 5 years	13	13.8
**Number of physicians trained to perform TnECHO in the unit (*n* = 94)**^a^		
1	49	52.2
2–4	35	37.2
> 4	10	10.6

*Abbreviations*: *NICU* neonatal intensive care unit, *PPHN* persistent pulmonary hypertension of newborn, *TnECHO* targeted neonatal echocardiography

^a^Number of responses received for that question

### Screening and diagnosis

It was observed that the three most common non-cardiac causes of PPHN were parenchymal lung diseases (97%), birth asphyxia (75%) and sepsis (52%); followed by respiratory distress syndrome (44%), congenital diaphragmatic hernia (16%) and pulmonary hypoplasia (2%). The most common predictor of PPHN was labile oxygen saturations (86%), followed by pre and post ductal saturation difference (77%) and high fraction of inspired oxygen (FiO2) requirements on respiratory support (73%) (Table 2). In units with facility for echocardiography, most commonly employed screening tools for PPHN were, echocardiography (80%), arterial blood gas (64%), oxygenation index (59%) and chest x-ray (41%). The specific echcardiographic criteria most commonly employed for diagnosing PPHN and its surveillance are outlined in Table 2.

**Table 2:** Screening and diagnosis

Characteristic	Responses N	Percentage%
**Most common predictors of PPHN (*n* = 118)**		
Labile oxygen saturations	101	85.5
Pre and post ductal saturation difference	91	77.1
High FiO2 requirements in a respiratory supported neonate	86	72.9
Discrepancy between chest Xray findings and degree of hypoxemia	46	39
**Most common screening tools for diagnosis of PPHN (*n* = 118)**		
Echocardiography	94	79.7
Arterial blood gas	75	63.6
Oxygenation Index	69	58.5
Chest X-Ray	48	40.7
**Most commonly utilized criteria for severity of PPHN (*n* = 118)**		
Echocardiography	92	78
FiO2 requirement	59	50
Oxygenation Index	54	45.8
**Specific echocardiographic criteria used to diagnose PPHN (*n* = 106)**^a^		
TR jet with PASP > 35 mmHg	52	51
Intra-ventricular septal flattening	51	50
Shunt pattern across PDA	49	48
TR jet with PASP > 25 mmHg	15	14.7
Intra-atrial shunt direction	14	13.7
Pulmonary artery acceleration time	13	12.7
**Frequency of echocardiography in a diagnosed case (*n* = 106)**^a^		
No fixed frequency	46	43.4
Daily until improvement	30	28.3
Alternate days until improvement	16	15.1
No follow up scan after diagnosis	14	13.2

*Abbreviations*: *PPHN* persistent pulmonary hypertension of newborn, *FiO2* fraction of inspired oxygen, *TR* tricuspid regurgitation, *PASP* pulmonary arterial systolic pressure, *PDA* patent ductus arteriosus

^a^Number of responses received for that question

### Management principles

**Fig. 2:**
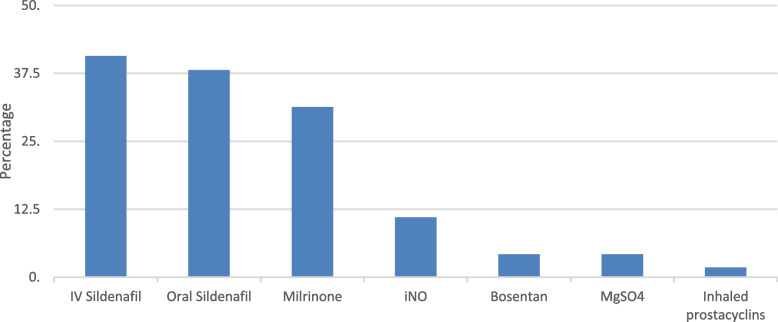
First line pulmonary vasodilator of choice

**Fig. 3:**
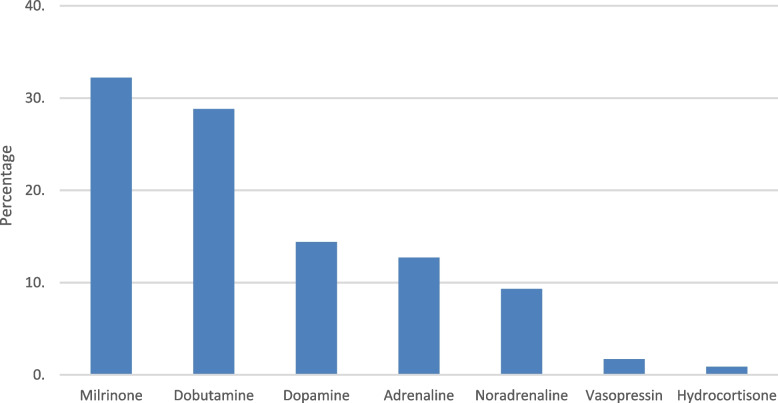
First line inotrope of choice

Around 81% units had standardized management guidelines being followed by all physicians, whereas 19% units reported that treatment strategies varied between physicians. Pre-ductal oxygen saturation targets and arterial oxygen pressure (PaO2) targets varied widely and are shown in Table 3. Out of the 99 responses received for use of paralytic agents, 54% units did not routinely paralyze patients of PPHN. Most common management strategies being followed were pulmonary vasodilators (88.1%), inotropes (85.6%), conventional ventilation (68.6%) and high frequency ventilation (59.3%). Around 59% used surfactant therapy. None of the participating units reported use of extracorporeal membrane oxygenation (ECMO). Most common indications for use of pulmonary vasodilators are shown in Table 3. Figure 2 depicts the order of preference of pulmonary vasodilators for PPHN; the most preferred being sildenafil. Other less commonly employed drugs were sodium bicarbonate, adenosine, L-arginine and intravenous (IV) prostaglandins. Around 25% of respondents reported use of iNO. In the setting where iNO was available, indications for starting iNO therapy, dosages, duration, weaning criteria and percentage of non-responders are listed in Table 3. Among units that used iNO, about 49% utilized it for babies with gestational age ≥ 35 weeks. However, many units used it for smaller preterm infants as well [32–34 weeks (24%), 28–32 weeks (17%) and < 28 weeks (10%)]. The order of preferred first line inotropic agent is depicted in Fig. 3, with milrinone being the most preferred.

**Table 3:** Management strategies

Characteristic	Responses N	Percentage %
**Pre-ductal oxygen saturation targets (*n* = 118)**		
90–94%	48	40.7
> 95%	29	24.6
92–96%	24	20.3
88–94%	17	14.4
**Target PaO2 in the acute phase (*n* = 118)**		
30–50 mmHg	03	2.5
50–70 mmHg	85	72
70–100 mmHg	28	23.7
100–120 mmHg	02	1.8
**Commonly used management strategies (*n* = 118)**		
Pulmonary vasodilators	104	88.1
Inotropes/ cardiotropes	101	85.6
Conventional ventilation	81	68.6
High frequency ventilation	70	59.3
Surfactant therapy	69	58.5
ECMO	0	0
**Indication for usage of pulmonary vasodilator therapy (*n* = 118)**		
Echocardiographic features of PPHN	54	45.8
High FiO2 requirement	46	39
High oxygenation index	18	15.2
**Most preferred indication for starting iNO therapy (*n* = 29)**^a^		
OI > 20 ppm	13	44.8
OI > 25 ppm	5	17.2
OI > 15 ppm	4	13.8
Failure of high frequency ventilation	5	17.2
Functional echocardiography	1	3.5
Arterial blood gas	1	3.5
**Starting dose of iNO (*n* = 29)**^a^		
20 ppm	20	69
5–20 ppm	8	27.6
> 20 ppm	1	3.4
**Maximum dose of iNO (*n* = 29)**^a^		
20 ppm	16	55.2
20–40 ppm	12	41.4
> 40 ppm	1	3.4
**Average duration of iNO (*n* = 29)**^a^		
< 12 h	02	6.9
12–24 h	08	27.6
24–48 h	12	41.4
> 48 h	07	24.1
**Criteria for weaning off iNO (*n* = 29)**^a^		
Rule of 60^b^	17	58.7
After FiO2 requirements decrease to half of initial	07	24.1
After TnEcho shows resolution of pulmonary hypertension	04	13.8
After pre-post ductal saturation difference disappears	01	3.4
**Percentage of non-responders to iNO (*n* = 29)**^a^		
< 10%	10	34.5
10–20%	08	27.6
20–40%	07	24.1
40–50%	04	13.8

*Abbreviations*: *PaO2* arterial oxygen pressure, *ECMO* extracorporeal membrane oxygenation, *PPHN* persistent pulmonary hypertension of newborn, *FiO2* fraction of inspired oxygen, *iNO* inhaled nitric oxide, *OI* oxygenation index, *ppm* parts per million, *TnECHO* targeted neonatal echocardiography

^a^Number of responses received for that question

^b^Inspired oxygen below 60%, Pa O2≥60 mmHg for 60 min

### Outcome and follow-up

The mortality rate of PPHN observed was < 10% at 58% units, 10–30% at 30%, 30–50% at 10% and > 50% at 2% NICUs. The commonest causes of mortality with PPHN were birth asphyxia (45%), meconium aspiration syndrome (35%), and associated cardiac disease (33%). Congenital diaphragmatic hernia (19%) primary PPHN (11%), sepsis (3%), hypoplastic lungs (2%) were other common causes. In 61% units, the post-discharge follow-up of babies diagnosed with PPHN was combined with neuro-developmental follow up schedule at 3, 6, 9 and 12 months, while 39% preferred routine weekly/monthly follow-up.

## Discussion

Our study population predominantly comprised of trained neonatologists with an experience of more than 3 years; from teaching as well as non-teaching hospitals; with an average capacity of 10–30 NICU beds; catering to both inborn and outborn patients. Most of the NICUs admitted upto 100 newborns per month, of which, on an average, 1–3 patients were diagnosed with PPHN.

We found parenchymal lung diseases to be the most common non-cardiac cause of PPHN followed by birth asphyxia and sepsis. This was consistent with findings of other observational studies on PPHN [9, 10].

The tools employed for screening and diagnosing the severity of PPHN were echocardiography, arterial blood gas, oxygenation index and FiO2 requirement, which were similar to findings of the international survey on PPHN by Nakwan et al. [7]. Echocardiography facilities for diagnosis of PPHN were available at 90% of the units in our study, which has improved from the 80% reported in a point of care ultrasound (POCUS) survey of Indian NICUs published in year 2019 [11]. However, only two-third of the echocardiography procedures were performed by neonatologists, pediatricians or neonatal trainees, the rest by cardiologists. Diagnosis and surveillance of PPHN requires round the clock availability of bedside TnECHO facility, and pediatric cardiology services are not available 24 h, 7 days a week in most Indian NICUs as per the previous POCUS survey [11]. This highlights the need for TnECHO training of treating neonatal physicians for prompt bedside detection and management of PPHN. In the international survey by Nakwan et al. involving 51 countries [7], 95% of the respondents reported use of echocardiography for the diagnosis of PPHN. However, two-third of these were high income countries. In the PPHN survey of Canada and Aus-NZ region [12], echocardiography was reported to guide treatment of PPHN by 80–92% of the respondents, but the use of neonatologist-performed echocardiography was only 9% in Canada and 50% in Aus-NZ region at the time of the survey (year 2012). However, these numbers are likely to have improved over the last decade. Deshpande et al. explored reasons for not having neonatologist performed POCUS services in Indian NICUs in their 2019 survey. The common reasons cited were non-availability of trained personnel and strict pre-conception and prenatal diagnostic techniques (PC-PNDT) Act (even relatively minor violations of which, are subject to prosecution and severe punishments). Accredited training program in POCUS on a national level, with integration of POCUS policies with the PC-PNDT Act, was recommended by authors of this survey to overcome these barriers [11].

While treating PPHN, majority of the NICUs accepted pre ductal saturation between 90–94% and target PaO2 of 50–70 mmHg. The survey responses of Nakwan et al. showed similar target values [7]. However, Alapati et al. in their US based survey noted that two-third of their respondents used higher saturation target > 95% and almost half targeted PaO2 > 80 mmHg, thus indicating wide variability in protocols for oxygen titration across the world [13].

The majority of NICUs in our survey followed unit based standardized management guidelines for PPHN. However, few NICUs did report that treatment strategies were variable between physicians within the same unit. Non-availability of iNO in many NICUs and multiple second line treatment options in absence of iNO, could be the reason for individual preferences and variations in management strategies. The common management strategies being followed by respondents in our study were pulmonary vasodilators and inotropes as mentioned by other surveys of PPHN. Most participant units had facilities for conventional ventilation and about two-third had facilities for high frequency ventilation as well. However, nonselective pulmonary vasodilators such as sildenafil were most commonly preferred and use of iNO was reported by only 25% of the respondents. None of the participating units reported use of ECMO. In contrast, 83% of participants in the international survey by Nakwan et al. (which predominantly included high income countries); reported use of iNO and one third had ECMO [7]. Surprisingly, in the Canada-Aus-NZ survey, despite availability of iNO, physicians reported high likelihood of use of non-selective pulmonary vasodilators. Escalating cost of iNO, non-responsiveness to iNO and limited availability of ECMO facilities were speculated to contribute to this by the authors; as also rigorous scrutinization of iNO usage by clinical service directors in Canadian NICUs; indicating that iNO cost is prohibitive even in developed countries [12]. There is no literature available currently that provides details of iNO availability in India. Observational studies have cited non-availability of medical grade iNO, cost and improper instrumentation for administration and monitoring of iNO, as reasons for lesser use of iNO in Indian NICUs. Cost of the consumables for gas delivery and monitoring systems is also prohibitive. Many Indian NICUs use purified industrial grade iNO, which is relatively more affordable than the medical grade iNO [14]. This is also reflected by the order of preferred pulmonary vasodilators used in Indian NICUs, the most preferably used being sildenafil (intravenous more than oral) followed by milrinone, iNO, bosentan and magnesium sulphate. In the studies by Nakwan et al. [7] and Shivananda et al. [12], the first line pulmonary vasodilators were iNO, oral sildenafil and milrinone in that order. Since none of our respondents reported use of ECMO for PPHN in neonates, it is difficult to comment on its utilization in Indian setting. However the ECMO scenario is changing in India, with its increasing availability in private sector multispecialty health care facilities and attempts at cost effective innovations [15].

First line inotropic agents for PPHN preferred by our survey respondents were milrinone, dobutamine, dopamine, adrenaline, noradrenaline, vasopressin and hydrocortisone in that order. However, the surveys by Naikwan et al. and Shivananda et al. reported dopamine to be the most preferred inotrope for PPHN associated hypotension, followed by dobutamine, adrenaline and noradrenaline [7, 12]. Evidence suggesting that dopamine increases pulmonary artery pressure relative to the systemic arterial pressure in neonates could be a reason for lesser use of dopamine in our survey [16]. Among other agents, one unit in our survey reported use of sodium bicarbonate infusion, one used L-arginine and one unit used adenosine as part of management of PPHN. Alkali use for PPHN is associated with increased requirement of ECMO and need for oxygen at 28 days [1] and its use in the post nitric oxide era has decreased worldwide as per other PPHN surveys [12]. L-arginine and adenosine have been reported to have a role in PPHN as alternative therapies; however, limited data exists for the efficacy in neonates [17, 18]. Shivananda et al. have mentioned use of adenosine for PPHN by 7% of their respondents [12].

In Indian NICUs where iNO was available, threshold for starting iNO therapy (Oxygenation Index (OI) > 20) was similar to thresholds used in other surveys. Majority of the units in our survey used 20 ppm as the starting as well as the highest dose of iNO while in the Canada-Aus-NZ region [12], 40% reported using doses above 20 parts per million (ppm) and as high as 80 ppm. Average duration of iNO therapy in our survey was 24–48 h, and most common criteria for weaning iNO were ‘rule of 60′ [19] and decrement of FiO2 levels to half of initial. Interestingly, the variability in weaning criteria of iNO is also related to the cost of therapy in some countries. For example, in Canada, weaning of iNO was reported to be more aggressive than in Aus-NZ region as iNO is billed on an hourly basis in Canada while in Aus-NZ region, billing is according to number of cylinders used [12]. Despite recommendations against the use of iNO in babies with gestational age < 35 weeks [20], around half of the units in our survey were using iNO for lower gestational ages as well, with around one-fourth units using it for infants < 32 weeks. The Canada-Aus-NZ survey also reported a high rate of usage of iNO in preterm infants less than 34 weeks of gestation (87%) [12]. We speculate that these preterm infants may have had refractory hypoxemia unresponsive to other pulmonary vasodilators. In our survey, the response rate to iNO was reported to vary between 50–90%. This was not explored in previous surveys of other countries.

Almost 90% NICUs in our study reported the mortality rate of PPHN to be < 30% with two third of these having mortality rate < 10%. Mortality rate was not studied in the previous surveys of PPHN. However, observational studies from two countries of South-Asian region have reported mortality associated with PPHN to range between 21–29% which is consistent with our survey results [5, 21]. Mortality rate reported in observational studies from high income countries is lower (7–15%) [3].

To our knowledge, this is the first nationwide survey undertaken to get a cross-sectional overview of current management practices used in Indian NICUs for PPHN. A noteworthy feature of this study was that, despite a fairly comprehensive questionnaire, most of the questions were answered by the respondents. Also, an option for free text was provided wherever relevant, which allowed participants to add their comments if their opinion or practices differed from the alternatives provided. There are several limitations to this study however. Although our response rate was 74%, majority of our respondent units were private hospitals and many sick patients are often not treated in these hospitals due to high treatment cost. The results therefore may not be reflective of the practices of all neonatologists in the country. Also, some answers may reflect a perceptive opinion of the respondent and may not be the true representation of actual clinical practice. Since we had to limit the number of questions, certain aspects could not be explored, such as, details of ventilation strategies, preferences of sedation and analgesic agents, mortality with respect to various modes of therapy and type of training received by physicians performing TnECHO. Our survey questionnaire did not include details of ECMO practices for management of PPHN in Indian NICUs. Also the responses from the survey reflected limited use of ECMO in our country. So this aspect of PPHN management could not be explored through our study.

## Conclusion

In this survey of PPHN management practices in Indian NICUs, we found wide variability in the diagnostic criteria, oxygenation targets and treatment modalities among different neonatal units. Non-selective pulmonary vasodilators are more widely used in Indian NICUs than iNO. Our findings indicate an urgent need for development of evidence based national guidelines for standardized management of PPHN, as per availability of resources in India. Since bedside echocardiography is a key resource required for timely diagnosis and surveillance of response to treatment, structured TnECHO training programs for neonatal physicians are required across the country. Inhaled nitric oxide being the most proven pulmonary vasodilator for management of PPHN, policy makers could undertake analysis for provision of iNO at lower cost, particularly to tertiary care and mid-level hospitals. Meanwhile, additional research on low cost alternative therapies to iNO in Indian settings might be helpful, while results on experimental therapies are awaited.

## Data Availability

The datasets used and/or analysed during this study are available from the corresponding author on reasonable request.

## Abbreviations

PPHN: Persistent pulmonary hypertension of the newborn
iNO: Inhaled nitric oxide
NICU: Neonatal intensive care units
TnECHO: Targeted neonatal echocardiography
FiO2: Fraction of inspired oxygen
PaO2: Arterial oxygen pressure
ECMO: Extracorporeal membrane oxygenation
IV: Intravenous
POCUS: Point of care ultrasound
PC-PNDT: Pre-conception and prenatal diagnostic techniques
OI: Oxygenation index
ppm: Parts per million
